# Minimally Invasive Direct Repair of Bilateral Lumbar Spine Pars Defects in Athletes

**DOI:** 10.1155/2013/659078

**Published:** 2013-04-30

**Authors:** Gabriel A. Widi, Seth K. Williams, Allan D. Levi

**Affiliations:** ^1^Department of Neurological Surgery and the Miami Project to Cure Paralysis, University of Miami Miller School of Medicine, Lois Pope Life Center, 1095 NW 14th Terrace (D4-6), Miami, FL 33136, USA; ^2^Department of Orthopaedics, University of Miami Miller, School of Medicine, Miami, FL 33136, USA

## Abstract

Spondylolysis of the lumbar spine has traditionally been treated using a variety of techniques ranging from conservative care to fusion. Direct repair of the defect may be utilized in young adult patients without significant disc degeneration and lumbar instability. We used minimally invasive techniques to place pars interarticularis screws with the use of an intraoperative CT scanner in three young adults, including two athletes. This technique is a modification of the original procedure in 1970 by Buck, and it offers the advantage of minimal muscle dissection and optimal screw trajectory. There were no intra- or postoperative complications. The detailed operative procedure and the postoperative course along with a brief review of pars interarticularis defect treatment are discussed.

## 1. Introduction

Isthmic spondylolysis occurs most commonly at L5. The cause of spondylolysis in these patients is repetitive stress of the pars interarticularis with subsequent microfracture, which in turn may lead to a bony defect and cause progressive spondylolisthesis in up to 25% of cases. In some patients progressive back and radicular pain ensue. Treatment typically begins with nonoperative modalities, with surgery indicated when there is no evidence of fracture healing and symptoms are persistent despite at least 3 months of treatment. Surgical options include decompression in those rare cases where radiculopathy is the chief complaint, lumbar fusion, and direct repair of the pars defect. Direct repair of pars defects has been used to treat young patients with healthy-appearing disks on MRI and no evidence of instability. The Buck's screw technique is a widely accepted method of direct pars repair [1, 2]. Recent modifications of this technique using endoscopic assistance [3] and minimally invasive image-guided [4] techniques have been reported. In this paper we describe three young, healthy, and active patients with symptomatic pars defects who failed non-operative management and were treated with a minimally invasive technique for direct pars repair.

## 2. Case Report 

**Figure 1:**
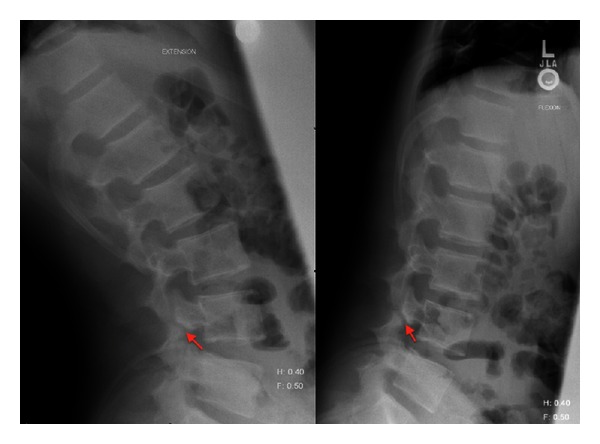
Flexion/extension preoperative rays of patient A showing increased separation of the pars defect with flexion.

**Figure 2:**
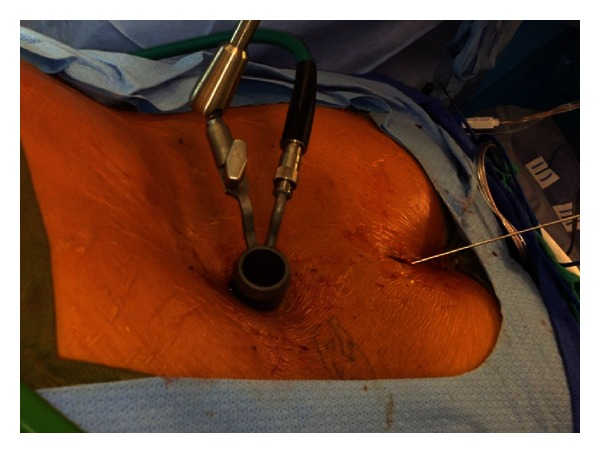
Intraoperative picture showing the METRx tube directed at the pars defect (left side of picture, which is towards the patient's head) and the guidewire for the screw (right side of the picture). In this case, a single midline cephalad incision was used to access both pars defects with the METRx tube, and a single midline caudal incision used the pass the guidewires and screws. Alternatively, two paired para-sagittal incisions can be used to access each pars defect and a single incision used to pass the guide wires and screws.

**Figure 3:**
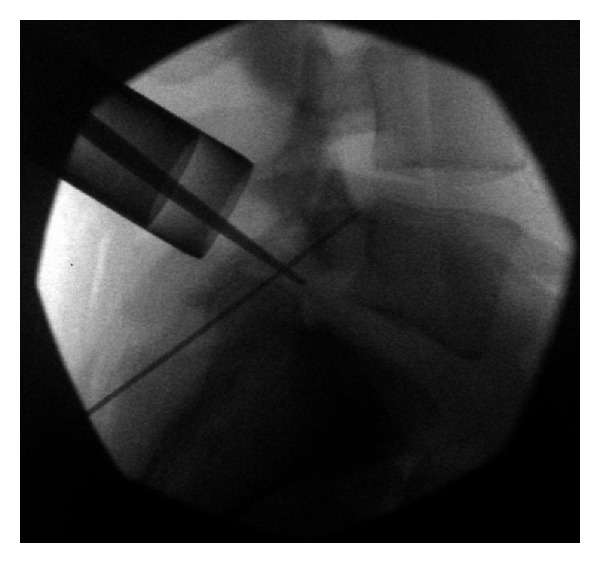
An 18 mm METRx tube was then directed towards the pars defect using fluoroscopy. The pars defect was decorticated with a high-speed drill, and local autograft and BMP were placed in the defect. Note that this step is done prior to placing the wire. This fluoroscopy image shows a currete in the pars defect after it is prepared with the bur.

**Figure 4:**
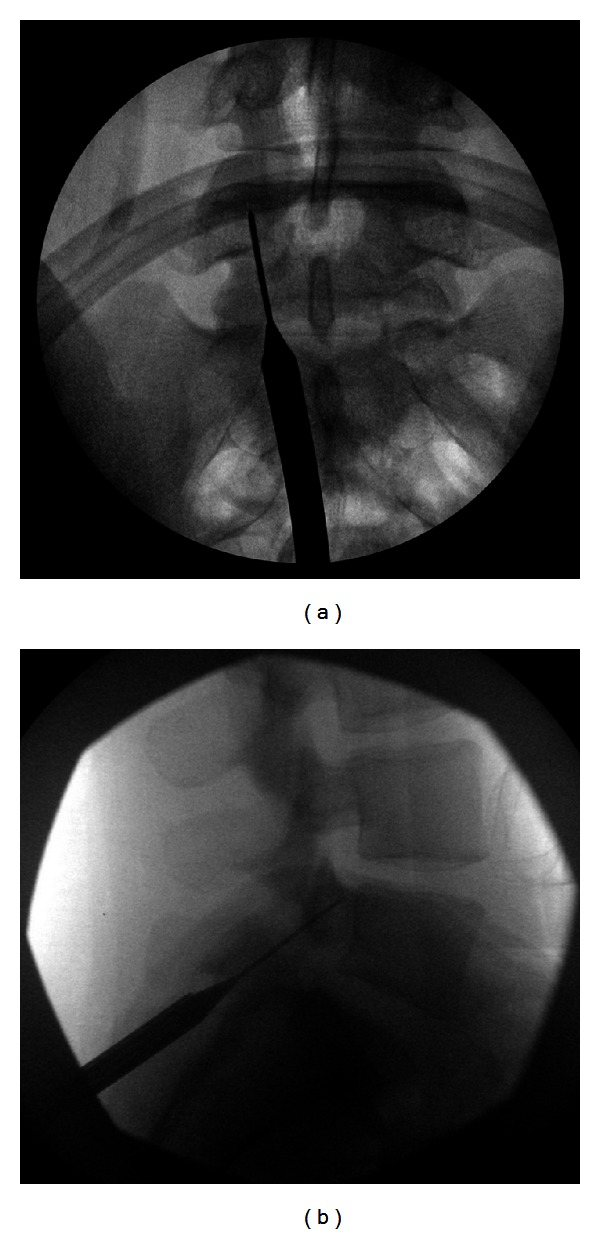
((a) and (b)) The UCSS cannula is advanced towards the undersurface of the lamina and then the guide wire drilled across the pars defect.

**Figure 5:**
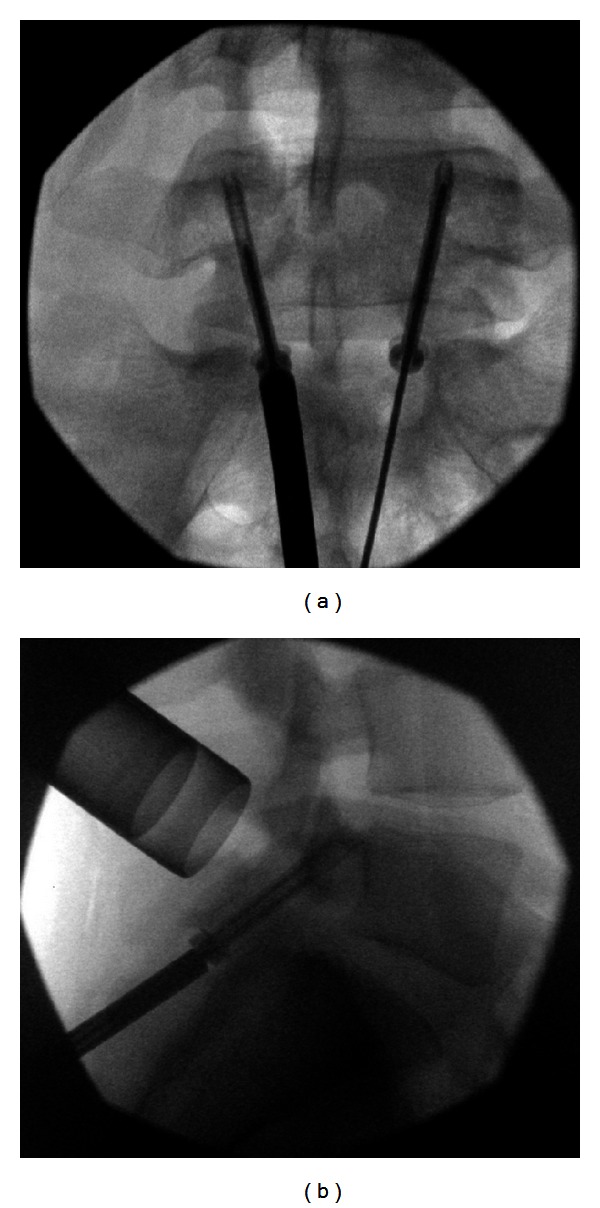
((a) and (b)) Cortical partially-threaded screws are passed across the pars defect.

**Figure 6:**
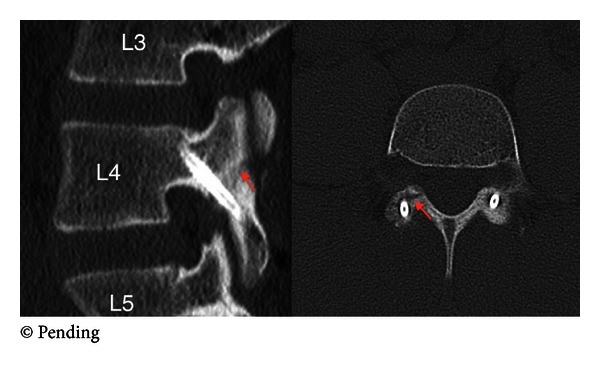
Three-month post-operative CT shows appropriate intraosseous screw placement with healing of the pars defect and no signs of nonunion.

Three young active patients presented with progressive back pain with plain imaging showing a bilateral pars defect (Table 1). An illustrative case is shown in Figures 1, 2, 3, 4, 5, and 6 of patient “A” with a bilateral pars defect at L4 treated via the described method. Initial treatment consisted of nonsteroidal anti-inflammatory medications, steroid injections, physiotherapy, bracing, and activity modification. These measures did not result in adequate healing of the fractures on followup, and symptoms were persistent.

## 3. Operative Technique in Detail

Biplanar fluoroscopy was used in conjunction with the Universal Cannulated Screw Set (UCSS; Medtronic, Memphis, TN, USA) and the METRx tubular table-mounted retractors (Medtronic). Lateral fluoroscopy was used to determine incision location by visualizing the trajectory required to access the pars defect. Either a single midline incision or paired parasagittal skin incision was made, followed by separate fascial incisions on both sides of the spinous process. Sequential dilators were placed under fluoroscopic guidance directed at the pars, and then an 18 mm diameter tubular retractor was placed in order to visualize the pars defects (Figure 2). The pars defects were identified, and the fibrous tissue debrided and a high-speed burr used to prepare the bony edges until the bleeding bone was encountered (Figure 3). 

Fluoroscopy was again used to identify the pars screw trajectory, and a separate single midline incision made in order to accommodate the UCSS cannula. The cannula was then tunneled through soft tissues and docked onto the inferior surface of the lamina, under fluoroscopic guidance. The guide wires were then drilled across the pars defects (Figures 4(a) and 4(b)). Once we were satisfied with the position of the wires, an intraoperative computed tomography (CT) scan was performed to confirm proper wire placement. We then drilled over the wires and placed 4 mm diameter partially threaded titanium cortical lag screws measuring 36–40 mm in length across the pars defects in order to achieve compression (Figures 5(a) and 5(b)). Prior to final tightening of the screws, one-half of a bone morphogenetic protein (BMP) sponge (Infuse; Medtronic) and bone shavings from the decortication were placed into each defect, and then final screw tightening was performed. The incisions were then closed in layers in the usual fashion. 

*Postoperative Course*. All three patients had minimal post-operative pain controlled by oral narcotics and were discharged home on post-operative day 1 or 2. Their incisions were well healed at the time of their first followup (10–14 days). CT scans obtained at three months post-operatively demonstrated definite healing of the pars fracture in two patients and evidence of early healing in one patient, satisfactory screw placement, and no signs of nonunion such as lucency around the screws (Figure 6). Flexion-extension imaging at three months as well showed no gap at the site of the pars defect and no spondylolisthesis. All patients have returned or are planning a return to their sport.

## 4. Discussion

Symptomatic isthmic spondylolysis is usually treated non-operatively with nonsteroidal anti-inflammatory medications, steroidal injections, physiotherapy with rigid braces, and avoidance of exacerbating injuries [5]. In Beutler et al.'s [6] seminal study analyzing the cause and progression of pars interarticularis defects in a group of 30 patients followed over a 45-year period, the authors demonstrated that the risk of spondylolisthesis progression in patients with bilateral interarticularis defects was similar to that of the general population. Further, they noted spondylolisthesis peaks in adolescence that diminished during adulthood. Other studies have reported similar findings [7–9], offering support for the relatively benign history of spondylosis, which would call for conservative nonsurgical management. In patients who have failed a trial of conservative therapy, there are several surgical options. 

Spinal fusion is not universally accepted given the resulting immobility across an otherwise healthy motion segment. Direct pars repair techniques were developed to address the defect and allow adjacent joint motion [10]. Kimura was the first to demonstrate this new concept in 1968 with pars interarticularis fixation without fusion of across adjacent facet joint [11]. Indeed, direct repair of a pars defect in symptomatic spondylolysis is a safe and effective option in young adults [6, 12]. Several techniques have been reported including the Buck's screw technique [1, 2, 13], a wiring method [14], and a pedicle screw and hook method [15]. Among these techniques, the Buck's screw method provides better biomechanical stability across the defect [16] and helps to restore physiological lumbar biomechanics altered by the pars defect [17]. This technique directs a lag screw from the ipsilateral lamina directly through the pars interarticularis defect, gaining purchase and stabilization of the lamina [18]. Using this method, Buck repaired the defect in 16 patients, with only one failure and two complications. The success rate of Buck's technique reported in the literature ranges from 67% to 93% [19–21].

Despite excellent outcomes and noting return to activity in 13 of 16 patients, this approach has not been widely popularized mainly due to a greater degree of technical difficulty compared to other techniques [7, 22]. This is despite the fact that the Buck's screw technique provides an attractive option to young active adults by limiting injury to the paraspinal muscles as occurs with posterolateral fusion techniques which cause subsequent paraspinal muscle atrophy and degeneration [23, 24]. A recent report described a minimally invasive approach using an endoscope to place these types of screws with iliac crest autograft in three young adults [3]. Similarly, image guidance was reportedly used to help fix the pars interarticularis defect in a young athlete [4]. Amoretti et al. [25] used a similar technique utilizing CT and fluoroscopy for percutaneous screw fixation for pars defects in adults under local anesthesia. In their study, 10 consecutive adult patients were prospectively treated by percutaneous screw fixation for low-grade isthmic spondylolisthesis of L5 with significant decrease in visual analog scale scores and Oswestry Disability Index measurements at two-year followup. Furthermore, neither slip progression nor screw failure was noted [25].

In the present study, the extra morbidity of iliac crest harvesting was avoided by using bone morphogenetic protein. We chose to use a half sponge in each of the defects based on the space available, but this dose has not been studied for this particular application. Other options would be to use autograft bone or demineralized bone matrix. The minimally invasive approach limits paraspinal muscle damage and optimizes early return to play while accomplishing a direct repair of the defect. In conclusion, a minimally invasive modification of the Buck's screw technique for direct repair of isthmic spondylolysis as presented here offers an excellent option to young active adults by minimizing muscle injury and preserving the adjacent joint.

## Figures and Tables

**Table 1:** 

Patient	Age	Duration of symptoms	Level	Duration of followup
A	17	13 months	L4	12 months
B	25	4 months	L4	7 months
C	20	8 months	L5	7 months
